# Comparison of 19 major infectious diseases during COVID-19 epidemic and previous years in Zhejiang, implications for prevention measures

**DOI:** 10.1186/s12879-022-07301-w

**Published:** 2022-03-28

**Authors:** Haopeng Li, Feng Ling, Shiyu Zhang, Ying Liu, Chongjian Wang, Hualiang Lin, Jimin Sun, Yinglin Wu

**Affiliations:** 1 Department of Epidemiology, School of Public Health, Sun Yat-Sen University, Guangzhou, Guangdong China; 2 Key Laboratory of Vaccine, Prevention and Control of Infectious Disease of Zhejiang Province, Zhejiang Provincial Center for Disease Control and Prevention, Hangzhou, Zhejiang China; 3 Department of Epidemiology and Biostatistics, College of Public Health, Zhengzhou University, Zhengzhou, Henan China

**Keywords:** Communicable diseases, COVID-19, Prevention and control

## Abstract

**Background:**

The global pandemic of coronavirus disease 2019 (COVID-19) has attracted great public health efforts across the world. Few studies, however, have described the potential impact of these measures on other important infectious diseases.

**Methods:**

The incidence of 19 major infectious diseases in Zhejiang Province was collected from the National Notifiable Infectious Disease Surveillance System from January 2017 to October 2020. The entire epidemic control phase was divided into three stages. The government deployed the first level response from 24 January to 2 March (the most rigorous measures). When the outbreak of COVID-19 was under control, the response level changed to the second level from 3 to 23 March, and then the third level response was implemented after 24 March. We compared the epidemiological characteristics of 19 major infectious diseases during different periods of the COVID-19 epidemic and previous years.

**Results:**

A total of 1,814,881 cases of 19 infectious diseases were reported in Zhejiang from January 2017 to October 2020, resulting in an incidence rate of 8088.30 cases per 1,000,000 person-years. After the non-pharmaceutical intervention, the incidence of 19 infectious diseases dropped by 70.84%, from 9436.32 cases per 1,000,000 person-years to 2751.51 cases per 1,000,000 person-years, with the large decrease in the first response period of influenza. However, we observed that the daily incidence of severe fever with thrombocytopenia syndrome (SFTS) and leptospirosis increased slightly (from 1.11 cases per 1,000,000 person-years to 1.82 cases per 1,000,000 person-years for SFTS and 0.30 cases per 1,000,000 person-years to 1.24 cases per 1,000,000 person-years for leptospirosis). There was no significant difference in the distribution of epidemiological characteristic of most infectious diseases before and during the implementation of COVID-19 control measures.

**Conclusion:**

Our study summarizes the epidemiological characteristics of 19 infectious diseases and indicates that the rigorous control measures for COVID-19 are also effective for majority of infectious diseases.

**Supplementary Information:**

The online version contains supplementary material available at 10.1186/s12879-022-07301-w.

## Background

In recent years, the implementation of various prevention and control measures has led to a significant reduction in some important infectious diseases and hence a reduction in the disease burden, the emphasis on infectious diseases has therefore largely diminished. However, the unprecedented global pandemic of coronavirus disease (COVID-19) reminds the threat of infectious diseases to human health [1]. It is estimated that 13–15 million deaths could be attributed to infectious diseases if the current infection level remains until 2030 [1]. More importantly, an increasing number of emerging infectious diseases is reported, for example, about 335 new infectious diseases emerged between 1940 and 2004 [2]. Therefore, precise prevention and control measures are needed to reduce the spread and burden of infectious diseases.

Various control measures have been implemented to mitigate the outbreak of COVID-19, such as quarantine of high-risk and sick persons, temporary or permanent closure of seafood and live poultry markets, increased teleworking, reduced operation in the service industry, early suspension of school and factory, the cancellation of mass gatherings, border control and public health education (including broader adoption of face masks and handwashing, hand and respiratory hygiene) [3–6]. Though these measures were initially specifically aiming for COVID-19, they might have significant impact on other infectious diseases. However, limited studies have been conducted to investigate the potential impacts of these measures on other infectious diseases [7–10]. Such evidence could provide important information for the control of other infectious diseases. We thus conducted this comparison study based on the incidence data of 19 common infectious diseases in Zhejiang Province, China from January 2017 to October 2020.

## Methods

### Study settings

Located in southeast of China, Zhejiang Province is composed of 2 sub-provincial cities and 9 prefecture-level cities. It has a land area of 105,500 square kilometers and a population of about 58.5 million residents by the end of 2019. It is an economically developed province in China with a relatively well public health system [11]. As a coastal province, it has a subtropical monsoon climate with an annual average temperature of 15–18 ℃.

### Data collection

The data were collected from the National Notifiable Infectious Disease Surveillance System (NNIDSS), which was established by the Chinese Government and covered 1.3 billion people from all regions in mainland China [12]. According the Law of the People's Republic of China on prevention and control of infectious diseases, the notifiable infectious diseases can be divided into three categories, Class A, Class B and Class C (see Additional file 1). Since January 20, 2020, the Corona Virus Disease 2019 (COVID-19), defined as Class B infectious diseases, should be taken measures as Class A infectious diseases [13].

We collected a total of 1,821,107 incident cases of 19 notifiable infectious diseases in Zhejiang Province, from January 2017 to October 2020. We extracted the information including sex, age, occupation, date of disease onset, and date of diagnosis. Due to the fewer cases of malaria and dysentery, we combined falciparum malaria, plasmodium vivax malaria, and other malaria into malaria; and combined bacterial and amoebic dysentery into dysentery. To ensure the accuracy of the data, we excluded the data with interval longer than 30 days from onset to diagnosis, and 1,814,881 cases remained.

### Period of the non-pharmaceutical interventions

**Fig. 1:**
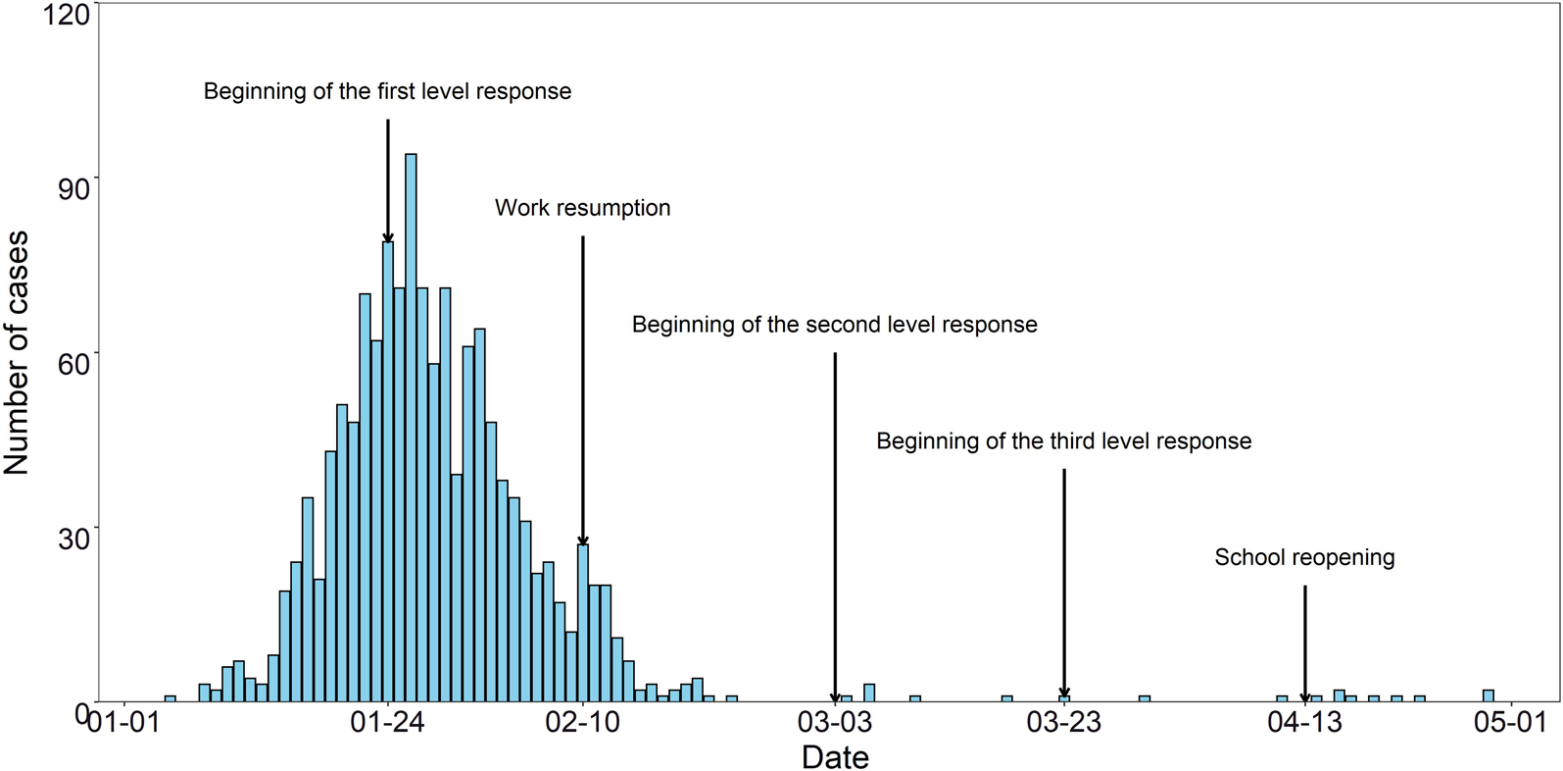
Number of COVID-19 cases from 1 January to 1 May in 2020

To control the outbreak of COVID-19, three levels of response measures have been implemented by Zhejiang Provincial People's Government. The first level response was implemented on 24 January, 2020, which was adjusted to the second level on 2 March [14, 15]. Finally, the government has carried out the third level since 23 March as the normalized prevention measures [16]. Because of the novel feature of the SARS-CoV-2, the epidemic prevention measures were mainly reflected in non-pharmaceutical interventions (NPIs). Figure 1 shows the specific events and the time when they were implemented.

In period of first level response, all the measures should be conducted under the leadership and command of the State Council. In the early stage of COVID-19, all localities focus on criteria that was internal non-proliferation and external non-export, making great efforts in closed-loop control and quantitative management, further improving the accuracy, effectiveness and coverage of control measures [14]. Besides, the important NPIs of first level response consisted of [14, 17]: (1) With advanced big data grid system, the strictest comprehensive investigation was carried out to effectively identify and cut off the source of infection; (2) Early detection, diagnosis and treatment of cases was implemented, as well as tracking management of the close contact, so as to identify and control the source of infection early; (3) The government made the adequate and force preparation on medical care and public health resources and deployed unified management to meet the basic needs of health care workers and residents for personal protection; (4) Health publicity has been strengthened, together with keeping social distances, temperature monitoring, wearing masks and hand-washing to protect the susceptible population; (5) A cordon sanitaire of Hubei province or high-risk regions were set up, and the residents who returned from other provinces need to stay at home at least 14 days, to reduce the opportunity of transmission of the virus with the mobility of people [18]; (6) To reduce the risk of exposure to the infection, all events where crowds were expected to gather were cancelled or postponed, including commercial events, entertainment venue, workplace and school reopening; (7) The Spring Festival holiday was extended to 2 February, work resumption was not as early as 9 February, and schools could be reopened in batches from 13 April, 2020 [19, 20]. Compared with the measures of first level response, the measures of second level response and third level response were relatively loose, mainly reflected in four aspects [15, 16, 21]. First, intercity travel restrictions were relaxed. In second level response, with health quick response code (health QR code), people only travelling from low-risk regions no longer needed to be tracing for 14 days, while the restrictions in Hubei province were further lifted in third level response, but still not in medium–high risk regions. Second, community restrictions were loosened. People should show health QR code, take their temperature and register the information before entering the community in period of second level response, while they could pass the community after showing their health QR code in the third level response. Third, the load capacity limitations on public transport and intercity travel were liberalized, raising the load capacity of ground public traffic from 50 to 75 percent, and the load capacity of rail transit from 50 to 65 percent in second level response. In period of third level response, public transport restrictions were released based on normalized prevention and control measures. Fourth, school reopened gradually after the epidemic of COVID-19 was under control (see Additional file 2).

### Statistical analysis

The time trend of monthly incidence density and city-specific incidence density was calculated to describe the spatiotemporal distribution of diseases. City-specific incidence density (per 1,000,000 person-years) was defined as the number of total incident cases that was multiplied by the length of observation in units of year, and then divided by the population size in different cities. In order to research the epidemiological characteristic of various diseases, we also described the distribution by sex, age and occupation, and analyzed the differences of distribution of age or occupation between male and female.

We then calculated the average daily incidence of various infectious diseases in the corresponding date of three level response to observe whether the incidence of each infectious disease changed under different epidemic prevention and control stages, such as the average daily incidence from 24 January to 2 March of 2017 to 2019 corresponding to the first level response. As well, we calculated the sex-specific proportion, age-specific proportion, occupation-specific proportion, and the mean value of interval from onset to diagnosis to assess the epidemiological feature variation of infectious diseases before and after the COVID-19 epidemic in different response levels. For quantitative variables, Wilcoxon test was used to determine whether the epidemiological characteristics of each infectious diseases before the outbreak of COVID-19 was significantly different from it after the outbreak, while Chi-square test was utilized for qualitative variables.

We conducted all the analyses with R software (version 3.6.1), and a two-sided *P* value of 0.05 was considered statistically significant.

## Results

### Descriptive results

Between January, 2017 and October, 2020, a total of 1,814,881 cases of 19 infectious diseases were reported, resulting in an average incidence of 8088.30 cases per 1,000,000 person-years (Table 1). The infectious diseases with the highest average incidence were influenza (3865.10 cases per 1,000,000 person-years), hand, foot, and mouth disease (HFMD) (2156.73 cases per 1,000,000 person-years) and other infectious diarrhea (1924.42 cases per 1,000,000 person-years), which together accounted for 98.24% (7946.25 of 8088.30 cases) of the overall incidence. Except for typhoid and scrub typhus, overall incidence of the 17 infectious diseases was highest among male individuals compared with females. The median time from onset to diagnosis of these 19 major infectious diseases was within 0–9 days, and most cases were found within their early stage of onset.

**Table 1:** Description of the total number of cases of major infectious diseases, average age, number of male and female cases and their ratio, and time interval

Disease	Number of cases 2017–2020 (n)	Sex		Median age	Mean Interval from onset to diagnosis
		Male (n, percent)	Female (n, percent)		
HFMD	483,934	284,218 (58.73)	199,716 (41.27)	2	1.04
Others	431,809	236,457 (54.76)	195,352 (45.24)	3	1.66
Dysentery	6966	3847 (55.23)	3119 (44.77)	25	1.51
Hepatitis e	6551	4004 (61.12)	2547 (38.88)	51	5.29
AHC	2990	1635 (54.68)	1355 (45.32)	36	1.39
Typhoid	705	351 (49.79)	354 (50.21)	42	6.72
Paratyphoid	243	132 (54.32)	111 (45.68)	26	6.25
Cholera	11	8 (72.73)	3 (27.27)	42	3.00
Dengue	2421	1360 (56.18)	1061 (43.82)	45	4.02
Scrub typhus	1310	617 (47.10)	693 (52.90)	59	5.03
Haemorrhagic fever	1212	877 (72.36)	335 (27.64)	51	6.41
Malaria	566	508 (89.75)	58 (10.25)	42	3.41
Brucellosis	373	269 (72.12)	104 (27.88)	51	9.59
SFTS	281	153 (54.45)	128 (45.55)	66	5.15
Leptospirosis	110	95 (86.36)	15 (13.64)	63.5	7.98
Rabies	33	30 (90.91)	3 (9.09)	51	4.00
Typhus	27	16 (57.14)	12 (42.86)	62	8.44
Influenza	867,265	444,107 (51.21)	423,158 (48.79)	8	1.24
Scarlet fever	8073	4854 (60.13)	3219 (39.87)	6	1.58
All	1,814,881	983,538 (54.19)	831,343 (45.81)	5	1.32

*HFMD* hand, foot, and mouth disease, *Others* infectious diarrheal diseases other than cholera, dysentery, typhoid, and paratyphoid, *AHC* acute hemorrhagic conjunctivitis, *SFTS* severe fever with thrombocytopenia syndrome

**Fig. 2:**
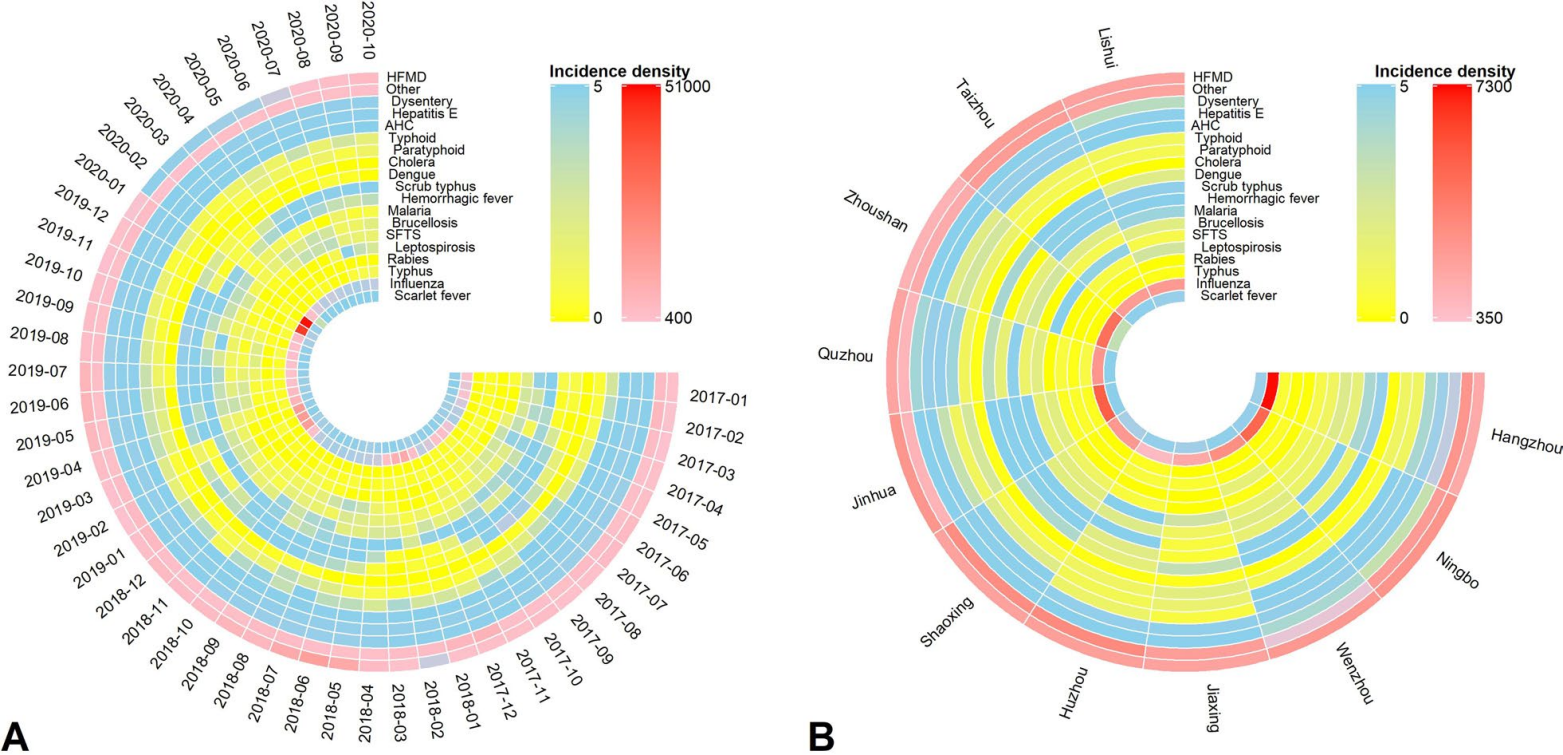
Spatiotemporal distribution of 19 infectious diseases (per 1,000,000 person-years). **A** Monthly incidence density of each infectious disease. **B** The total incidence density of each infectious disease in 11 cities. A circle represents a disease, and a radial column represents a month (left) or one city (right). *HFMD* hand, foot, and mouth disease, *Others* infectious diarrheal diseases other than cholera, dysentery, typhoid, and paratyphoid, *AHC* acute hemorrhagic conjunctivitis, *SFTS* severe fever with thrombocytopenia syndrome

Most of infectious diseases were characterized by seasonal distribution, such as dysentery (May to September), scrub typhus (June) and influenza (January) (Fig. 2). In the whole province, HFMD presents an annual summer and winter peak; other infectious diarrheal diseases showed a semi-annual activity peak, including a major peak in winter, followed by a minor peak in summer. Haemorrhagic fever and scarlet fever show annual peak activity in summer and autumn, and SFTS also presents a semi-annual activity peak, a major peak in summer, then a minor peak occurred in autumn. Overall, the total incidence density of 8 infectious diseases varied greatly between cities in Zhejiang Province, while the total incidence of the remaining 10 infectious diseases varied less widely between different cities or too few cities. Taizhou, Hangzhou, Jinhua, Ningbo and Wenzhou are the most frequent cities with the high incidence of 19 infectious diseases. Among the 19 cities with the highest incidence of infectious diseases, Hangzhou appears most frequently (Fig. 2).

**Fig. 3:**
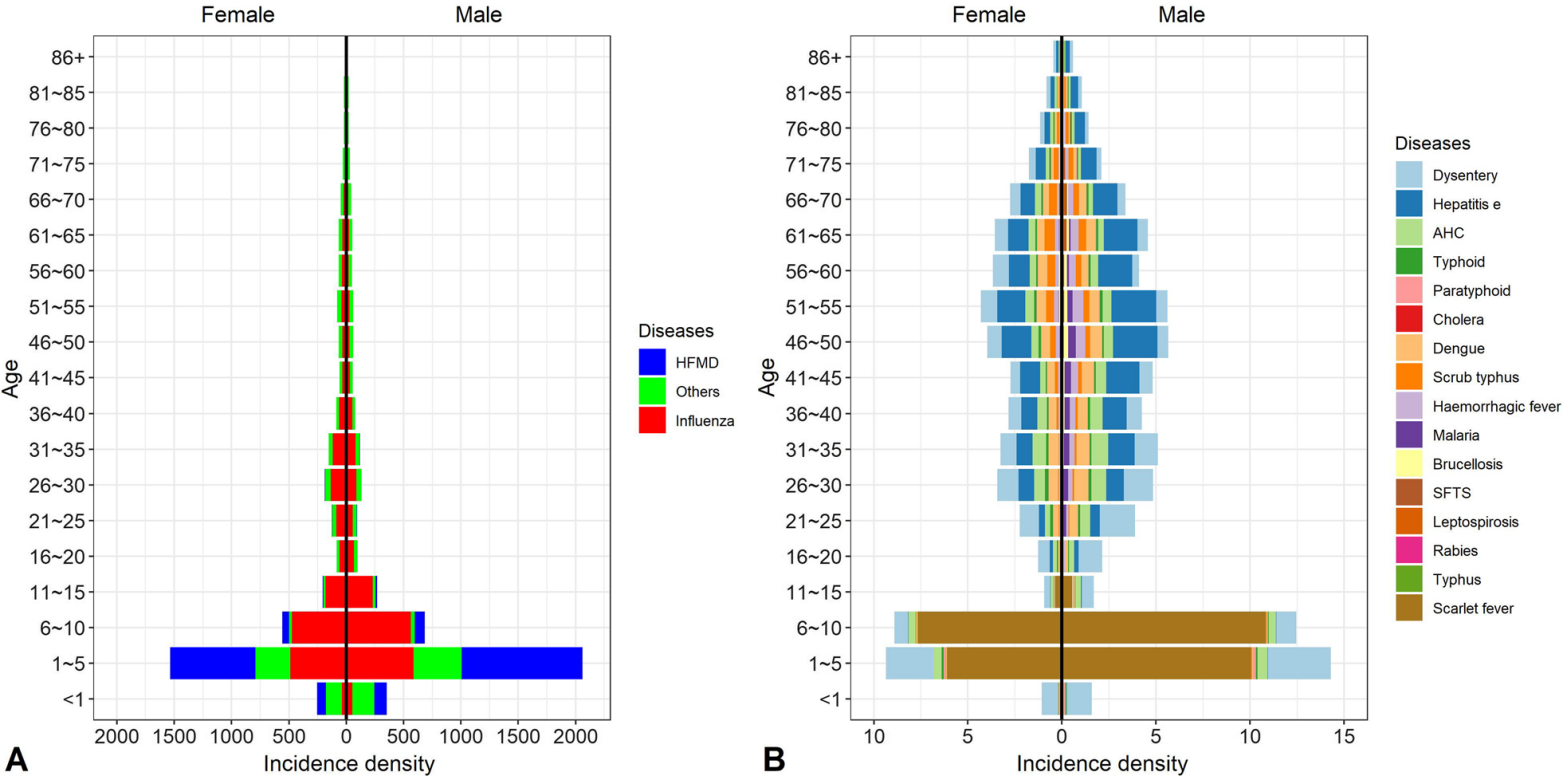
Incidence density of each disease by sex and age (per 1,000,000 person-years). **A** The incidence of HFMD, other infectious diarrhea and influenza. **B** The overall incidence of the remaining 16 infectious diseases. Age was divided into groups every 5 years, ranging from less than 1 year old to over 85 years old. The left side of the dotted line is female and the right side is male. *HFMD* hand, foot, and mouth disease, *Others* infectious diarrheal diseases other than cholera, dysentery, typhoid, and paratyphoid, *AHC* acute hemorrhagic conjunctivitis. *SFTS* severe fever with thrombocytopenia syndrome

**Fig. 4:**
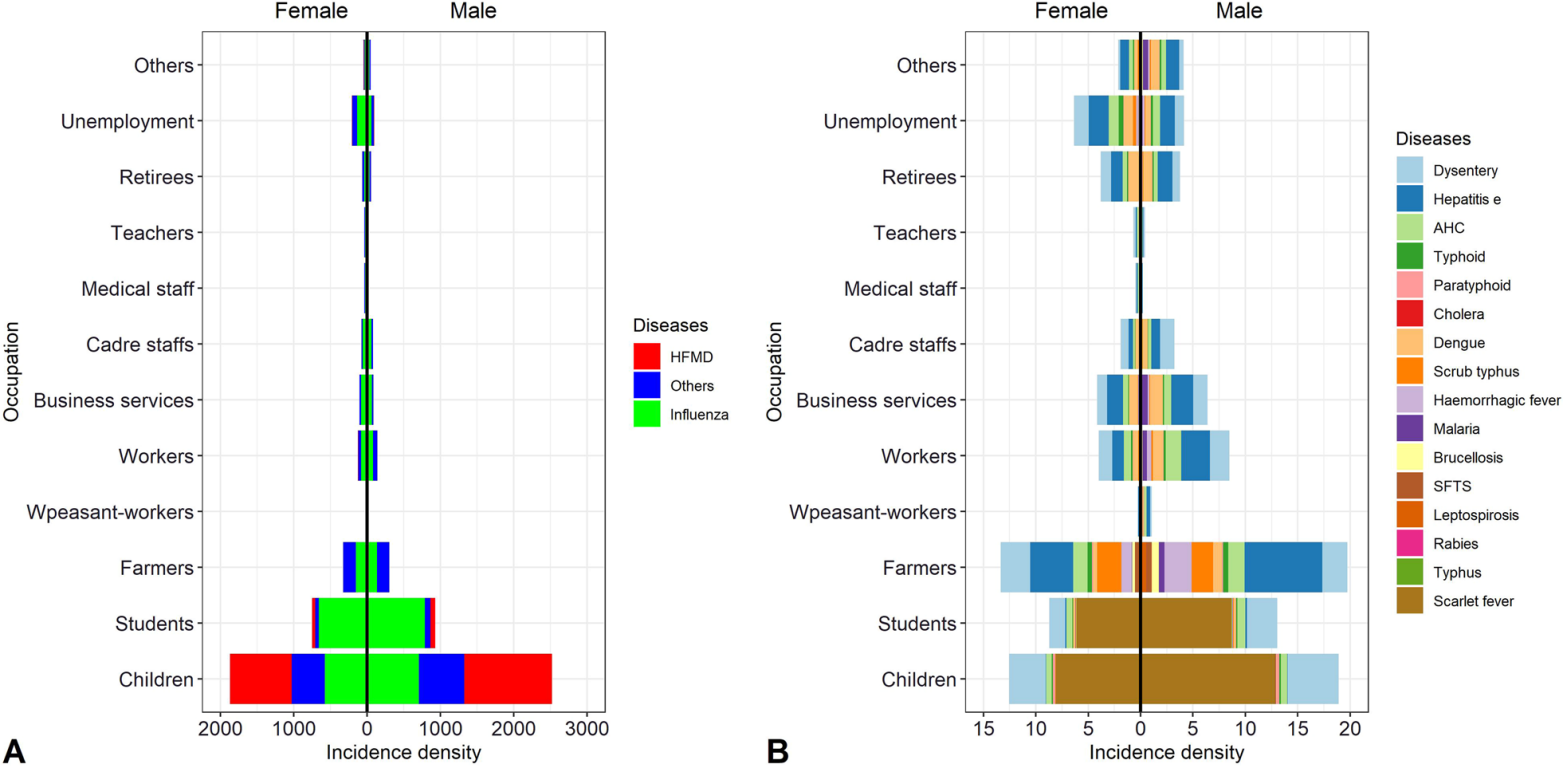
Distribution of each disease by sex and occupation (per 1,000,000 person-years). **A** The incidence of HFMD, other infectious diarrhea and influenza. **B** The overall incidence of the remaining 16 infectious diseases. It divides occupations into children, students, farmers, peasant-workers, workers, business services, cadre staffs, medical staff, teachers, retirees, unemployment, others. The left side of the dotted line is female and the right side is male. *HFMD* hand, foot, and mouth disease, *Others* infectious diarrheal diseases other than cholera, dysentery, typhoid, and paratyphoid, *AHC* acute hemorrhagic conjunctivitis, *SFTS* severe fever with thrombocytopenia syndrome

In different age groups, the sex-specific distribution was similar, the overall incidence among males was slightly higher than that among females. In different genders, the incidence of HFMD, other infectious diarrheal diseases, influenza and scarlet fever was highest among children younger than 10 years. 15 of 19 infectious diseases rhombic distributed in young and middle-aged group, except for SFTS, leptospirosis and typhus in the elderly. The incidence of 19 infectious diseases was highest among children aged 1–10 years, and lowest among the elderly aged over 75 years (Fig. 3). In different occupational group, overall incidence among male individual was higher than that among female, except for unemployment, teachers, medical staff and retirees. The incidence of 19 infectious diseases was highest among children aged 1–10 years, and lowest among the elderly aged over 75 years. Among the adult occupation, farmers had a highest incidence, followed by workers. Especially, overall incidence of scrub typhus, haemorrhagic fever, brucellosis, SFTS, and leptospirosis was higher among farmers (Fig. 4).

### Comparison of the incidence of major infectious diseases before and after COVID-19

Since the COVID-19 was reported in January 2020, the incidence of five of 19 infectious diseases has declined precipitously, such as HFMD, hepatitis E, malaria, influenza, and scarlet fever, while the incidence of typhoid and paratyphoid became more stable after COVID-19 (Table 2). However, the incidence of SFTS and leptospirosis rose slightly. The contemporaneous incidence of paratyphoid and dengue fever did not statistically differ before and after the secondary response, and the contemporaneous incidence of scrub typhus, SFTS and rabies only statistically significantly differed before and after the third response. Besides, there was no significantly difference found in the contemporaneous incidence of leptospirosis and typhus before and after the response. The contemporaneous incidence of remaining 12 communicable diseases had statistically significant differences (*P* < 0.05) before and after the each of 3 level measures. In addition, the average daily incidence of 5 infectious disease increased as the relaxation of the policy. And the minimum incidence of other infectious diseases occurred in secondary response, which may be related to the lag of policy effect.

**Table 2:** Incidence density in different diseases in three period of policy (per 1,000,000 person-years)

Diseases	Common	Policy 1	Common	Policy 2	Common	Policy 3
HFMD	383.53	45.88*	493.01	30.97*	3543.92	732.51*
Others	3240.18	775.96*	2352.43	625.02*	1644.84	1480.64*
Dysentery	19.52	9.79*	17.26	13.14*	40.97	30.89*
Hepatitis e	36.53	16.68*	39.72	16.58*	31.02	21.32*
AHC	11.41	15.40	10.29	4.69*	15.98	8.47*
Typhoid	2.99	0.96*	3.64	0.63*	4.11	1.85*
Paratyphoid	0.27	0.16	0.42	0.31	1.73	0.93*
Cholera	0.00	0.00	0.00	0.00	0.08	0.06*
Dengue	1.33	0.80	0.21	0.00	21.05	0.08*
Scrub typhus	0.11	0.64	0.00	0.63	8.24	8.56*
Haemorrhagic fever	5.39	0.64*	4.37	2.82*	5.02	4.52*
Malaria	2.77	1.12*	2.39	0.63*	3.24	0.65*
Brucellosis	1.65	0.96*	1.66	2.19*	1.77	1.99*
SFTS	0.00	0.16	0.00	0.31	1.69	2.24*
Leptospirosis	0.00	0.00	0.00	0.00	0.48	1.57
Rabies	0.27	0.16	0.21	0.00	0.18	0.00*
Typhus	0.00	0.00	0.00	0.00	0.12	0.31
Influenza	8030.37	4565.90*	6050.37	245.88*	878.34	138.68*
Scarlet fever	18.02	5.45*	27.97	6.88*	39.68	7.46*

*representing the difference between the policy compared to the same period is significant.

*HFMD* hand, foot, and mouth disease, *Others* infectious diarrheal diseases other than cholera, dysentery, typhoid, and paratyphoid, *AHC* acute hemorrhagic conjunctivitis, *SFTS* severe fever with thrombocytopenia syndrome

**Fig. 5:**
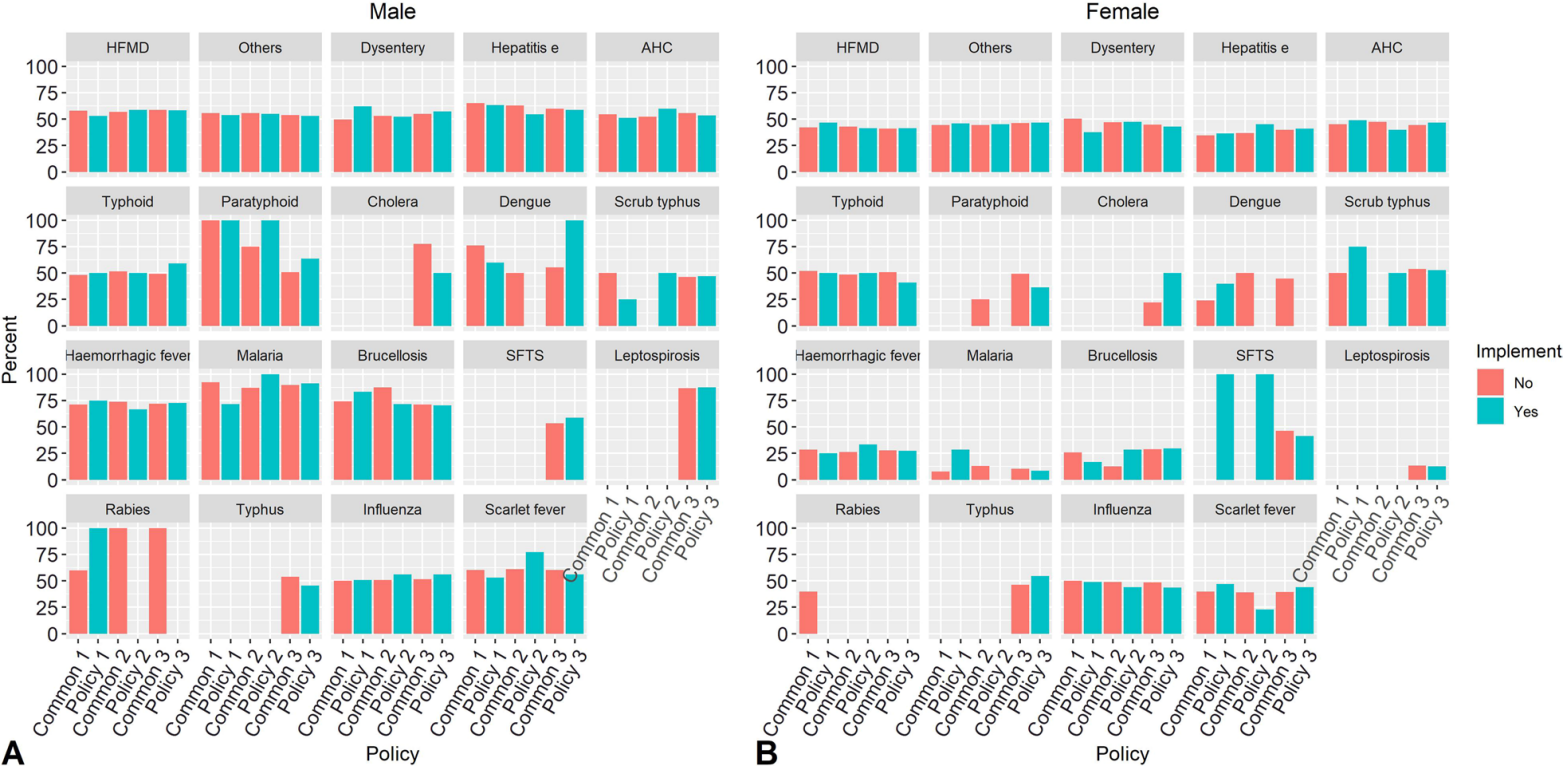
Contemporaneous comparison of the incidence of major infectious diseases in male (**A**) and female (**B**) before and after COVID-19. 17–19 Period 1 was from 24 January to 2 March in 2017, 2018, and 2019. 17–19 Period 2 was from 3 to 22 March in 2017, 2018, and 2019. 17–19 Period 3 was from 23 March to 31 October in 2017, 2018, and 2019. 20 Period 1, 20 Period 2, and 20 Period 3 were in the same way. *HFMD* hand, foot, and mouth disease, *Others* infectious diarrheal diseases other than cholera, dysentery, typhoid, and paratyphoid, *AHC* acute hemorrhagic conjunctivitis, *SFTS* severe fever with thrombocytopenia syndrome

Similarly, we used the same method to assess whether the contemporaneous gender-specific incidence of 19 infectious diseases was different before and after the tertiary response, and found only the contemporaneous gender-specific incidence of influenza showed statistically significant difference before and after each level of response (*P* < 0.05), with the increased proportion of male individual. At the same time, the contemporaneous gender-specific incidence of other infectious diarrhea showed statistical difference before and after the primary and tertiary responses, and the proportion of male decreased. While there were no statistically differences in the contemporaneous gender-specific incidence of the other 17 infectious diseases before and after the implementation of each level of measures (Fig. 5).

We observed a significant difference of proportion of 7 infectious diseases before and after the response implement (Table 3). Among them, the age-specific proportion of HFMD, influenza and other infectious diarrheal diseases was all significantly different in three level of response. The proportion of elder than 5 years in HFMD and other infectious diarrheal diseases was on the rise, while it was opposite in influenza, dysentery, scarlet fever and typhoid.

**Table 3:** The statistical significantly distribution of age of different diseases with policy compared the same period

Diseases	Implement	< 1	1 ~ 5	6 ~ 10	11 ~ 15	16 ~ 20	21 ~ 25	26 ~ 30	31 ~ 35	36 ~ 40	41 ~ 45	46 ~ 50	51 ~ 55	56 ~ 60	61 ~ 65	66 ~ 70	71 ~ 75	76 ~ 80	81 ~ 85	86 +
AHC	Common	0	8	4	4	6	11	**12**	8	6	7	6	5	7	5	4	2	2	2	0
	Policy 3	0	4	2	2	**10**	7	9	9	6	6	7	8	2	8	7	6	4	2	1
Dysentery	Common	8	**17**	3	6	10	9	7	5	4	4	5	4	5	4	3	2	1	1	1
	Policy 3	7	**22**	3	6	10	9	6	4	3	3	4	4	8	3	3	2	1	1	1
HFMD	Common	7	**84**	1	0	0	0	0	0	0	0	0	0	7	0	0	0	0	0	0
	Policy 1	13	**72**	2	0	0	1	0	0	0	0	0	0	11	0	0	0	0	0	0
	Common	6	**87**	1	0	0	0	0	0	0	0	0	0	6	0	0	0	0	0	0
	Policy 2	25	**59**	4	0	0	1	0	0	0	1	0	0	10	0	0	0	0	0	0
	Common	9	**83**	1	0	0	0	0	0	0	0	0	0	7	0	0	0	0	0	0
	Policy 3	9	**85**	1	0	0	0	0	0	0	0	0	0	4	0	0	0	0	0	0
Influenza	Common	4	**33**	4	3	4	7	6	4	3	4	4	3	12	3	2	1	1	1	1
	Policy 1	4	**30**	3	3	6	11	9	5	2	3	3	2	11	2	2	1	1	1	1
	Common	3	**32**	9	3	4	5	4	3	2	2	2	2	24	2	1	1	1	1	0
	Policy 2	5	**26**	4	5	3	6	4	4	2	4	4	3	6	4	4	4	3	3	4
	Common	3	**26**	7	4	5	9	7	3	2	2	2	2	22	2	1	1	1	1	1
	Policy 3	4	**37**	6	4	3	3	3	2	2	2	2	2	12	3	3	3	2	2	2
Others	Common	17	**56**	1	1	2	3	2	1	1	2	2	2	2	2	1	1	1	0	0
	Policy 1	15	**42**	2	2	2	4	3	3	2	3	4	3	3	4	3	2	1	1	1
	Common	18	**50**	2	2	2	3	2	2	2	2	3	2	3	2	1	1	1	1	0
	Policy 2	17	**32**	2	1	5	4	3	2	2	3	4	4	7	3	3	2	2	1	1
	Common	17	**22**	2	4	5	7	5	4	4	4	5	4	3	4	3	2	2	1	1
	Policy 3	14	**19**	3	5	6	7	6	4	4	4	5	5	4	4	4	3	2	2	1
Scarlet fever	Common	0	**49**	3	0	0	0	0	0	0	0	0	0	48	0	0	0	0	0	0
	Policy 2	0	**73**	9	0	0	0	5	0	0	0	0	0	14	0	0	0	0	0	0
	Common	1	**49**	3	0	0	0	0	0	0	0	0	0	48	0	0	0	0	0	0
	Policy 3	1	**51**	5	1	1	0	0	0	0	0	0	0	40	0	0	0	0	0	0
Typhoid	Common	2	2	0	0	9	**21**	9	9	4	7	11	4	4	5	5	4	4	2	0
	Policy 1	0	0	0	**33**	0	0	17	0	0	0	0	0	0	17	**33**	0	0	0	0

Bold represent the maximum proportion

*AHC* acute hemorrhagic conjunctivitis, *HFMD* hand, foot, and mouth disease, *Others* infectious diarrheal diseases other than cholera, dysentery, typhoid, and paratyphoid

Chi-square tests were performed for the contemporaneous occupational incidence of 19 infectious diseases before and after the tertiary response: the contemporaneous occupational incidence of other infectious diarrhea, influenza, and HFMD were statistically significant before and after each level of response (*P* < 0.05) (Table 4). No statistically difference was observed in terms of contemporaneous career-specific incidence of AHC only before and after the second level response. A statistically difference was observed in terms of contemporaneous career-specific incidence of scarlet fever, hepatitis e, malaria and brucellosis only before and after the first level response, and of dysentery only before and after the third level response. And we could observe that the proportion of farmers in brucellosis has descended, also in malaria, and the proportion of workers or unemployment was increased. While the proportion of farmers in AHC has increased.

**Table 4:** The statistical significantly distribution of career of different diseases with policy compared the same period

Diseases	Implement	Business services	Cadre staffs	Children	Farmers	Medical staff	Others	Retirees	Students	Teachers	Unemployment	Workers	Peasant-workers
AHC	Common	7	4	7	**22**	1	8	7	7	4	13	18	1
	Policy 1	4	6	4	**44**	3	2	5	4	1	8	11	6
	Common	9	3	9	**21**	1	6	6	12	1	11	18	3
	Policy 3	7	1	5	**23**	3	8	11	4	1	19	18	1
Brucellosis	Common	10	3	0	**61**	0	10	0	0	0	13	3	0
	Policy 1	0	0	0	17	0	33	0	17	0	0	**33**	0
Dysentery	Common	8	8	**26**	16	1	2	6	14	1	8	10	1
	Policy 3	9	9	**31**	14	1	1	4	15	1	6	9	0
Hepatitis e	Common	9	5	0	**45**	1	8	9	0	1	8	12	0
	Policy 1	8	2	0	**42**	3	5	7	1	0	18	13	1
HFMD	Common	0	0	**93**	0	0	0	0	6	0	0	0	0
	Policy 1	0	0	**88**	0	0	0	0	10	0	0	1	0
	Common	0	0	**96**	0	0	0	0	4	0	0	0	0
	Policy 2	1	0	**85**	1	0	0	0	13	0	0	0	0
	Common	0	0	**95**	0	0	0	0	5	0	0	0	0
	Policy 3	0	0	**95**	0	0	0	0	4	0	0	0	0
Influenza	Common	5	4	**40**	14	2	2	4	15	1	8	6	0
	Policy 1	6	4	**36**	15	2	3	2	14	1	8	8	0
	Common	3	3	**39**	9	1	2	2	31	1	6	4	0
	Policy 2	3	1	**32**	31	2	4	3	11	1	6	7	0
	Common	5	4	**33**	10	1	2	2	28	1	7	5	0
	Policy 3	2	2	**43**	17	2	2	2	20	0	5	3	0
Malaria	Common	21	2	0	**42**	0	13	0	6	0	2	13	0
	Policy 1	14	0	0	14	0	14	0	0	0	**43**	14	0
Others	Common	1	1	**74**	11	0	0	1	4	0	3	3	0
	Policy 1	2	1	**58**	20	1	1	2	5	0	5	4	0
	Common	1	1	**69**	12	0	1	2	6	1	3	3	0
	Policy 2	2	2	**51**	20	1	2	3	9	0	6	4	1
	Common	3	3	**40**	24	1	2	3	8	1	7	7	1
	Policy 3	4	3	**34**	25	1	2	3	10	1	9	8	1
Scarlet fever	Common	0	0	**67**	0	0	0	0	32	0	0	0	0
	Policy 1	3	0	**74**	3	0	0	0	21	0	0	0	0

Bold represent the maximum proportion

*AHC* acute hemorrhagic conjunctivitis, *HFMD* hand, foot, and mouse disease, *Others* infectious diarrheal diseases other than cholera, dysentery, typhoid, and paratyphoid

The Wilcoxon test revealed that the difference in interval in most of infectious diseases before and after the response was smaller than 1 day. No statistically difference was observed in terms of contemporaneous interval of others infectious diarrhea and dysentery only before and after the first level response, and a statistically difference was observed in terms of contemporaneous career-specific incidence of typhoid and scarlet fever only before and after the first level response, and of HFMD, AHC and influenza only before and after the third level response. While there were no statistically differences in the contemporaneous interval of the other 12 infectious diseases before and after the implementation of each level of measures (Table 5).

**Table 5:** Mean interval from onset to diagnosis in different diseases in three period of policy

Diseases	Common	Policy 1	Common	Policy 2	Common	Policy 3
HFMD	1.41	1.60	1.46	1.69	0.96	1.48*
Others	1.42	1.53	1.40	1.93*	1.79	1.91*
Dysentery	1.66	1.90	1.55	0.90*	1.49	1.48*
Hepatitis e	5.67	7.25	4.72	6.08	5.09	5.26
AHC	1.08	2.08	0.96	0.67	1.45	1.36*
Typhoid	7.55	13.83*	7.03	6.00	6.57	6.27
Paratyphoid	7.00	5.00	6.00	3.00	6.00	8.00
Cholera	0.00	0.00	0.00	0.00	2.78	4.00
Dengue	6.68	5.00	5.00	0.00	4.01	3.33
Scrub typhus	0.00	7.00	0.00	5.00	5.03	5.28
Haemorrhagic fever	8.04	12.75	6.90	8.78	5.93	6.60
Malaria	3.29	4.43	2.65	14.50	3.56	2.57
Brucellosis	10.52	14.17	10.69	11.14	8.54	10.48
SFTS	0.00	8.00	0.00	6.00	5.20	5.13
Leptospirosis	0.00	0.00	0.00	0.00	9.92	5.82
Rabies	3.60	3.00	4.50	0.00	4.16	0.00
Typhus	0.00	0.00	0.00	0.00	0.00	11.36
Influenza	1.58	1.10	1.25	1.52	1.61	1.53*
Scarlet fever	1.45	1.18*	1.45	1.95	1.52	1.75

*representing the difference between the policy compared to the same period is significant

*HFMD* hand, foot, and mouth disease, *Others* infectious diarrheal diseases other than cholera, dysentery, typhoid, and paratyphoid, *AHC* acute hemorrhagic conjunctivitis, *SFTS* severe fever with thrombocytopenia syndrome

## Discussion

It is analyzed that the incidence of some infectious diseases is different before and after the occurrence of COVID-19, and the characteristics of some of the diseases are also different. After the discovery of COVID-19 in January 2020, compared with before, the incidence of leptospirosis (especially men) and SFTS increased, however, the incidence of HFMD, hepatitis e, malaria, influenza and scarlet fever was down. Specifically, the incidence of typhoid and paratyphoid stabilized, and that of scrub typhus and hemorrhagic fever did not change. After measures of controlling the epidemic was implemented by stages in 2020, on the basis of contemporary comparison during 2017 to 2019, the total incidence of 12 of the 19 infectious diseases showed difference before and after the implementation, specifically, that of scrub typhus, SFDS and rabies had a difference between the contemporary comparison to the implementation of the tertiary response. The gender-specific incidence of 17 of the 19 infectious diseases had no difference, which showed that the effect of measures had no effect on the sexual morbidity of most infectious diseases. The career-specific incidence of 10 of the 19 infectious diseases also had no difference, but the effect of measures had an effect on the occupational morbidity of other infectious diarrhea, influenza and scarlet fever which are more common in children. And the interval between onset and diagnosis of 12 of the 19 infectious diseases also had no difference before and after the implementation. This study is expected to reduce the incidence of major infectious diseases and protect the vulnerable population of other infectious diseases while implementing prevention and control of new infectious diseases.

There has been a study showing a decrease in the incidence of enterovirus infections, influenza, scarlet fever [22] and this is in line with our study, and the reason may be that this observation is related to the implementation of actions and policies regarding COVID-19, such as early vigilance and aggressive measures to prevent the spread of droplets and contacts in public places and schools [22]. Hand hygiene can prevent common respiratory and gastrointestinal infections, especially HFMD. One of the measures is frequent hand washing to prevent transmission by contact, which also happens to reduce the incidence of HFMD [23]. The prevention and control measures, such as regular washing of public areas and surfaces, frequent hand washing and virtual meetings, have also prevented the spread of enteroviruses such as hepatitis E transmitted by the fecal–oral route, leading to a reduction in their incidence[24]. Early peaks and sudden decreases in influenza activity by the end of January 2020 were time-correlated with the social distancing effects of the government's COVID-19 mitigation strategy[25]. These findings suggest that certain community mitigation measures may be a useful complement to influenza vaccination during influenza season, especially for those at highest risk of severe illness or complications. Take the decreased incidence of influenza as an example. Initially, the decrease in influenza virus activity was attributed to the reduction in testing, as people with respiratory symptoms are usually given priority for COVID-19 assessment and testing. Second, other factors, such as a sharp decline in global travel or an increase in vaccine use, may have played a role in reducing the spread of influenza; However, the effect of these factors have not been assessed. Third, viral interference may help explain the absence of influenza during a pandemic caused by another respiratory virus, which may be more competitive than influenza in the respiratory tract[9].

The diagnosis, epidemiology, clinical manifestation, laboratory performance and treatment of acute febrile diseases (AFI) including dengue fever, leptospirosis, typhoid fever, malaria, scrub typhus are similar to COVID-19, which may be misdiagnosed or only found one of the two (such as ignoring AFI due to the pandemic of COVID—19 now), resulting in delays in diagnosis and treatment, expanded spread, rise of its incidence and an extended interval between cases detected (this is the same as our observations), because there are similarities, which can be in control of the COVID-19 and relieve the symptoms of AFI, that reminds us to be vigilant with the diseases of overlapping symptoms[26]. Current measures to control COVID-19 include isolation of suspected cases, isolation of infected patients, contact tracing and other strategies. These quarantine measures may not be easy to accept for people in some countries or regions. As a result, many patients with clinical characteristics similar to COVID-19 and/or any other disease with similar symptoms may be hesitant to voluntarily seek testing and treatment in a formal health facility. Even if contact tracing is critical to stopping transmission, some patients may have privacy concerns [27]. Therefore, the COVID-19 response should be adjusted for such underserved and vulnerable populations in accordance with local social, epidemiological and economic profiles to prevent the emergence of similar infectious diseases [27].

For zoonotic diseases such as malaria and scrub typhus, although our observations of malaria (decreased or unchanged) are inconsistent with the literature (increased) [28], this suggests that effective prevention and control measures implemented early in the epidemic period can effectively reduce the burden of the disease. In the case of malaria, bed nets need to be updated regularly to prevent malaria, but the distribution has been delayed or canceled due to outbreaks. In terms of detection and treatment of malaria, individuals may stop accessing health-care facilities due to fear of exposure to COVID-19 or inability to afford transportation, and health-care workers need additional resources to protect themselves against COVID-19[28]. Previous study predicted that the disruption to malaria control in Africa associated with COVID-19 could nearly double mortality of malaria by 2020 and could lead to further increases in mortality in subsequent years [29]. However, some studies have shown that early intervention with COVID-19 could effectively reduce the scale of the epidemic and its impact on malaria transmission potential [30]. A more comprehensive COVID-19 response that minimizes indirect deaths from malaria is therefore warranted [31].

According to the nature, damage degree and range of public health emergencies, it is divided into four levels which are general (IV), large (III), major (II) and special major (I). Different responses have been adopted at different levels of governments. Targeted prevention and control will be carried out by the unit of counties (districts), in different areas and at different levels. On the basis of comprehensive analysis and judgment of population and incidence, scientific classification of epidemic risk levels will be made to formulate prevention and control strategies. This is conducive to avoiding "one-size-fits-all" prevention and control measures and "convergence" management regardless of regional and industrial differences. Regional and tiered epidemic prevention and control can make it more targeted, precise, scientific and effective, minimize the impact of the epidemic on economic and social development and people's lives, and coordinate epidemic prevention and control with economic and social development. The prevention and control of the epidemic cannot be loosened, but the normal order of production and life and the legitimate demands of enterprises for resuming production and work should be respected, and the livelihood and convenience of the people should be guaranteed. At present, with the positive achievement in the prevention and control work, the situation of the epidemic had undergone positive changes, and the epidemic has entered a new stage of focusing on prevention and control and development at the same time. The shortening of the emergency response stage also means that the region has started to make tentative preparations for resuming work and production from the policy level. In addition to the control of the epidemic, the resumption of work and production is being carried out in an orderly manner. Low-risk areas need to adjust their strategies to prevention of foreign imports as soon as possible, so that production and living order can be fully restored. Medium-risk areas need to resume work and production in an orderly manner based on the situation of prevention and control. High-risk areas need to continue to focus on prevention and control of the epidemic. It is worth noting that the adjustment of response level does not represent a turning point of the epidemic. The current situation of the epidemic is still grave and complex, and the prevention and control of the epidemic is in the most critical stage.

The current study had a few limitations. First, the ecological analysis cannot establish a causal association, although we observed a consistent result across the studies. Second, our assessment may be limited by the application of retrospective and descriptive methods that involve the analysis of publicly available monitoring data. Third, there may have been changes in the way certain diseases are diagnosed between 2017 and 2020, which may have influenced our results. Fourth, because the COVID-19 pandemic has been fluctuating, it is difficult to obtain data on other infectious diseases after the epidemic has completely subsided and to compare the situation of other infectious diseases before, during and after the outbreak. Finally, the transmission rate of some infectious diseases in Zhejiang Province has been low in the past few years, such as typhoid, paratyphoid, scrub typhus, hemorrhagic fever, malaria, SFTS, leptospirosis, and typhus, and it is hard to observe real changes in their incidence due to the small number of those cases. And little research has been done on changes in the incidence of these infectious diseases after the outbreak.

## Conclusion

In conclusion, we comprehensively describe the epidemiological characteristics of infectious diseases and analyze the difference before and after the epidemic of COVID-19 in Zhejiang Province. The overall incidence of most of infectious diseases has been a precipitous decline since the response implemented, while cases of some infectious diseases increased, such as leptospirosis and SFTS. The incidence increased with the relaxation of the policy, and the characteristic of some infectious diseases has been changed after the implement of response measure. Therefore, stricter prevention and control measures of infectious diseases should be taken, with a focus on children, male individual and farmers.

## Supplementary Information

**Additional file 1.** The classification of 21 infectious diseases.

**Additional file 2.** The non-pharmaceutical interventions of different level.

## Data Availability

The datasets generated and/or analysed during the current study are available in the GitHub repository, https://github.com/HollyLee9601/Data-of-manuscript-on-BMC-ID.git.
