# Supplementary material for: Comparison of 19 major infectious diseases during COVID-19 epidemic and previous years in Zhejiang, implications for prevention measures
Source: BMC Infect Dis. 2022 Mar 28;22:296. doi: 10.1186/s12879-022-07301-w (PMC8958816; doi:10.1186/s12879-022-07301-w)
Supplement: Supplementary file 1 — Additional file 1. The classification of 21 infectious diseases. [file 12879_2022_7301_MOESM1_ESM.docx]

**Additional file 1.** The classification of 21 infectious diseases

Category A infectious disease: cholera.

Category B infectious disease: hepatitis e, haemorrhagic fever, rabies, dengue, bacterial dysentery, amoebic dysentery, typhoid, paratyphoid, scarlet fever, brucellosis, leptospirosis, malaria, COVID-19.

Category C infectious disease: influenza, acute haemorrhagic conjunctivitis (AHC), typhus, hand, food, and mouth disease (HFMD), infectious diarrhoeal diseases other than cholera, bacterial dysentery, amoebic dysentery, typhoid, and paratyphoid.

Others: severe fever with thrombocytopenia syndrome (SFTS), scrub typhus.
