# Supplementary material for: Comparison of 19 major infectious diseases during COVID-19 epidemic and previous years in Zhejiang, implications for prevention measures
Source: BMC Infect Dis. 2022 Mar 28;22:296. doi: 10.1186/s12879-022-07301-w (PMC8958816; doi:10.1186/s12879-022-07301-w)
Supplement: Supplementary file 2 — Additional file 2. The non-pharmaceutical interventions of different level. [file 12879_2022_7301_MOESM2_ESM.docx]

**Additional file 2.** The non-pharmaceutical interventions of different level

| Level and time | Measures |
| --- | --- |
| First level  (2020.01.24-03.02) | - Early detection, diagnosis and treatment of cases - Centralized quarantine of the suspected cases and closely contacts - Restriction of public gathering activities - Promotion of personal protective measures - Postponement of the Lunar New Year holidays - Strengthening of disinfection in public |
| Second level  (2020.03.03-03.22) | - Opening intercity travel for low-risk cities in mainland China, but restriction for the high-risk cities and Hubei Province - Unlocking the restriction of the healthy person gradually, but implement the administration of access registration, and still closure of the public amenities - Raising the ratio of traffic load, but only 75% for ground traffic and 65% for rail traffic - Permitting the open for campus in small size, and monitoring the physical condition of students |
| Third level  (2020.03.23-10.31) | - Unlocking the restriction for Hubei Province, but not for high-risk area and area beyond the borders - Opening the closure of the public amenities gradually - Opening the public transport system, but still strictly implementing the disinfection - Resuming the normal classes at all grades |
